# Comparison of the Effects of Abdominal Massage and Oral Administration of Sweet Almond Oil on Constipation and Quality of Life among Elderly Individuals: A Single-Blind Clinical Trial

**DOI:** 10.1155/2022/9661939

**Published:** 2022-06-21

**Authors:** Amir Faghihi, Mohammad Ebrahim Zohalinezhad, Majid Najafi Kalyani

**Affiliations:** ^1^School of Nursing, Fasa University of Medical Sciences, Fasa, Iran; ^2^Department of Persian Medicine, Shiraz University of Medical Sciences, Shiraz, Iran; ^3^School of Nursing and Midwifery, Shiraz University of Medical Sciences, Shiraz, Iran

## Abstract

**Background:**

Constipation is a common digestive disorder in the elderly population, which has a considerable impact on various aspects of their lives. Traditional and complementary medicines are two common treatments for constipation. This study was aimed at comparing the effects of abdominal massage and oral administration of sweet almond oil on constipation and quality of life among elderly people.

**Methods:**

In this single-blind study, 90 eligible elderly people were randomly selected according to ROME IV diagnostic criteria from October 2020 to May 2021 and were divided into three groups using block randomization (*n* = 30). In the oral consumption group, 126 ml sweet almond oil was administered during seven sessions held over two weeks (18 ml every other day). The abdominal massage group was provided with abdominal massage with the same amount of sweet almond oil during seven sessions in two weeks. Finally, the third group (i.e., the control group) received no interventions. Constipation was measured using the Constipation Assessment Scale (CAS) and the *Bristol* Stool Form *Scale* (BSFS) at baseline and on the 15^th^ day of the intervention. Patient Assessment of Constipation-Quality of Life (PAC-QOL) was also applied before and after the intervention (day 15). The data were entered in to the SPSS 22 software and were analyzed using chi-square *t*-test, paired *t*-test, independent *t*-test, and ANOVA. *p* ≤ 0.05 was considered statistically significant.

**Results:**

The results revealed a significant decrease in the CAS score in the oral consumption group (i.e., oral administration of sweet almond oil; from 7.81 ± 2.62 to 1.15 ± 1.08, *p* < 0.0003) compared to the abdominal massage group (i.e., abdominal massage; from 8 ± 2.57 to 2.26 ± 1.81, *p* < 0.0002) and the control group (from 7.73 ± 2.53 to 6 ± 2.74, *p* < 0.0002). Additionally, the stool consistency score in BSFS was significantly higher in the oral consumption group in comparison to the two other groups (*p* < 0.0003) before and after the intervention. Furthermore, the quality of life score decreased more significantly in the oral consumption group (from 75.04 ± 7.66 to 28.15 ± 3.30, *p* < 0.0003) than in the abdominal massage group (from 81.15 ± 5.15 to 43.26 ± 4.13, *p* < 0.0001) and the control group (from 79.62 ± 4.76 to 62.31 ± 6.40, *p* < 0.0008).

**Conclusion:**

Since the oral administration of sweet almond oil and abdominal massage was highly influential in the participants' constipation scores and quality of life, these cost-effective methods with low complication rates are recommended to be used alongside other treatments in managing constipation among older adults.

## 1. Background

Considering the improvement of population control and treatment methods followed by the increase in life expectancy in the recent decades, the world's population has started aging [1]. Aging may cause numerous physical, cognitive, and social problems for elderly individuals and affect their quality of life [2, 3]. Several common age-related diseases include cardiovascular disease, chronic lung disease, diabetes, and constipation due to prolonged physical inactivity [4, 5]. Evidence has indicated that approximately 25% of elderly people suffered from constipation, as one of the most common digestive disorders [6]. It has also been shown that the prevalence of constipation increased with age [7]. Several studies performed in Iran demonstrated that the prevalence of constipation ranged from 3.5% to 32.9% [8, 9].

Constipation can be a symptom or a disease, and it is necessary to be examined due to its long-term complications as well as its impact on the quality of life [10]. Older adults experience severe constipation with more complications due to their physiological changes associated with aging, inactivity, excessive use of medicines, hypotension, and inattention to the disease [11]. The prevalence of constipation in this population has a negative impact on their quality of life and welfare. The decrease in elderly individuals' quality of life in relation to constipation can manifest through headaches, fatigue, abdominal pain and bloating, loss of appetite, nausea and vomiting, and worsening of other symptoms such as limb spasticity and bladder dysfunction [12, 13].

A wide range of pharmacological and nonpharmacological techniques can be utilized to manage and treat constipation in elderly individuals. Pharmaceutical methods expose patients to high expenses and numerous side effects [14]. Therefore, nonpharmacological methods such as traditional and complementary medicine have become popular in the recent decades [15]. In complementary medicine, vegetable oils play a vital role in preventing and treating constipation [16]. In Persian medicine, vegetable oils extracted from extra virgin olive, sweet almond, and violet have been administered topically and orally to treat various diseases such as constipation without fever [17, 18].

Persian medicine has been used in management of various diseases. A study by Nayebi et al. showed that Melissa officinalis was effective in decreasing the serum level of triglyceride in diabetic patients [19]. In Persian medicine text book, several treatment principles are mentioned for the management of catarrh [20] and opium addiction [21]. Furthermore, a study by Rahmani et al. showed that Nigella sativa oil was effective in reducing oxidative stress, inflammation, and blood sugar in diabetic patients [22].

Almond is rich in healthy fats, proteins, minerals, and vitamins, and its oral and topical administration can help prevent and treat various diseases [23]. Sweet almond oil is one of the almond extracts whose effectiveness in skin diseases such as pruritus and itchy skin, reduction and prevention of stretch marks, *striae* distensae, and fatty acids has been proven [24–26]. Similar to laxatives, sweet almond oil can stimulate intestinal nerves to induce bowel movements and transit due to its cholinergic effects [27]. It can also act as a probiotic to increase gut microbiota, particularly intestinal bacteria, resulting in the treatment of constipation [28].

On the other hand, several studies have referred to abdominal massage as another cheap and noninvasive nonpharmacological method for controlling and treating constipation [29, 30].

In Persian medicine, almond oil has been introduced as a treatment approach for constipation [31, 32]. In a study by Schincaglia et al., almond oil improved bowel habit in patients [33]. Improving in bowel habits is related to lifestyle and quality of life [34].

Considering the high prevalence of constipation in the elderly population and its effect on their quality of life, the limited number of studies on the effect of sweet almond oil on constipation in older adults, and studies on abdominal massage using sweet almond oil to control and treat constipation in Persian medicine, the present study was aimed at comparing the effects of abdominal massage and oral administration of sweet almond oil on constipation and quality of life among elderly people in Shiraz. We hypothesized that constipation would relieve after applying abdominal massage and using oral of sweet almond oil. Furthermore, we hypothesized that by improving in bowel habit, the quality of life would improve in elderly.

## 2. Materials and Methods

### 2.1. Study Design

This single-blind study was conducted in the gastroenterology department of geriatric clinics in Shiraz, Iran, from October 2020 to May 2021. Based on a previous study [35], considering the constipation score and error of 1%[*ὰ* = 0.01), power of 90%[*β* = 0.10), *μ*1 = 3.73, *μ*2 = 2.57, *σ*1 = 1.83, and *σ*2 = 0.85, and comparison of two means in MedCalc software, 26 people were estimated for each study group. However, considering 15% attrition, 30 individuals were assigned to each group using the following formula:

(1) $$\begin{array}{c} n=\frac{{\left(Z_{1-a/2}+Z_{1-b}\right)}^{2}\left(\sigma_{1}^{2}+\sigma_{2}^{2}\right)}{{\left(\mu_{1}-\mu_{2}\right)}^{2}}. \end{array}$$

### 2.2. Subjects

The inclusion criteria of the study were aging over 60 years, obtaining at least two scores based on the ROME IV diagnostic criteria or one or more scores based on the Constipation Assessment Scale, and being willing to cooperate. The individuals who suffered from comorbidities, cognitive impairment (i.e., dementia), and diarrhea before and during the study; had a history of abdominal surgery, abdominal hernia (i.e., inguinal and umbilical hernias), and mechanical or pseudoobstruction; had inflammation and open wound around the massage area; had skin conditions, diseases, and scars on the abdomen; and were unwilling to use oil were excluded from the study.

Based on the ROME IV criteria, 124 elderly patients with functional constipation referred to the gastroenterology department of geriatric clinics in Shiraz were randomly selected. The ROME IV criteria are evaluated based on some indicators such as defecation less than three times a week, lumpy stools, straining, feeling of obstruction in the rectum, feeling of incomplete evacuation, and manual help to facilitate defecation, which are valid for patients with two or more indicators. Finally, 34 patients were excluded from the study, and 90 eligible patients were examined.

### 2.3. Intervention

Before the beginning of the study, the procedure was explained to the participants and their written informed consent forms were obtained. Then, the participants were randomly divided into three groups; i.e., oral administration of sweet almond oil, abdominal massage with sweet almond oil, and control group (*n* = 30 in each group), via permuted block randomization (ABC) using random number generator. Group A was allocated to the oral administration of sweet almond oil, group B was assigned to abdominal massage with sweet almond oil, and group C was the control group. The numbers were randomly selected from 1 to 90 and were then randomly assigned to each study group.

In the oral consumption group, 9 ml of sweet almond oil was administered orally every 12 hours (i.e., 18 ml daily), which was continued for seven sessions held every other day over two weeks. It is worth mentioning that the researchers conducted follow-up telephone calls every day. In the abdominal massage group, the patients were provided with abdominal massage at a depth of 2 cm with 18 ml of sweet almond oil for 15 minutes at specific times (i.e., 8-11 A.M.). This was done through seven sessions held every other day over a two-week period. In both groups, the sweet almond oil was the same in terms of chemical composition and substance concentration. The sweet almond oil was used in 18 mm packages, which were produced by Mehrabani Company under license No. 99912 issued by the Food and Drug Administration. In the first two sessions, a skilled and trained masseur performed abdominal massage in a geriatric clinic. Then, he agreed to provide the service in the patients' houses. Additionally, the researchers conducted follow-up telephone calls every day. The massage was performed based on the Abdominal Massage Quick Reference Guide proposed by McClurg et al. [36], which included the following nine steps:

Step 1. Stroke upwards to relax the abdominal muscles. In case of hiatus hernia or reflux, stroke down

Step 2. Stroke from the lumbar to stimulate the vagus nerve, which tells the bowel to wake up. Stroke from small of back, round and down inside of iliac crests. Finish stroke at the groin. Do ten strokes

Step 3. Effleurage (toothpaste stoke). Do this in a clockwise direction to stimulate bowel directions to move fecal matter along. Do this stroke for two minutes

Heart of massage, the kneading helps to propel the fecal matter along the colon to load the rectum.

Step 4. Palmar kneading, descending colon (down pipe) for two minutes

Step 5. Palmar kneading, up ascending colon (up pipe) for two minutes

Step 6. Repeat step 4 (down pipe) for two minutes

Step 7. Repeat step 3 for two more minutes

Step 8. Stroking to relax abdominal muscles and to help the body understand that the massage is ending. Do this ten times

Step 9. Vibrations over umbilicus to relieve flatus (wind). Do this four times [36]

**Figure 1:**
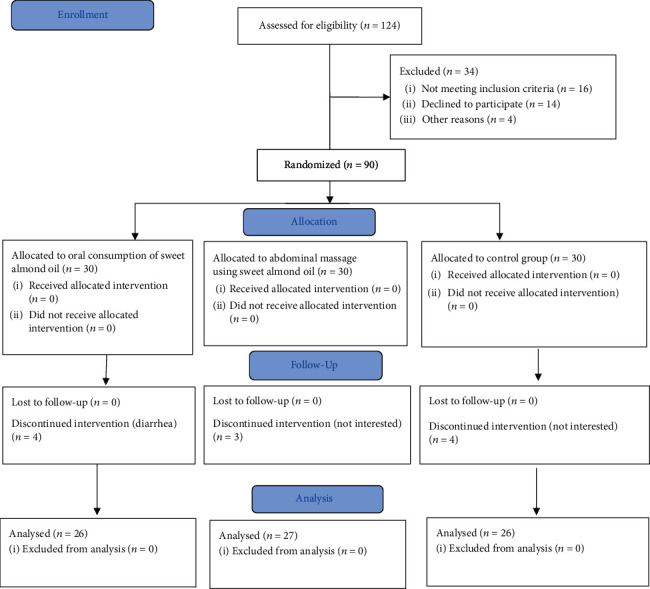
CONSORT diagram of the patients' progress through the stages of the randomized controlled trial.

The control group received no interventions. Patients in all the three groups received the medications prescribed by their physicians, and none of their medications were removed or lowered during the study (Figure 1).

### 2.4. Measurement

Before the intervention and on the 15^th^ day, the participants' constipation and quality of life were measured by the Constipation Assessment Scale (CAS), Bristol Stool Form Scale (BSFS), and Patient Assessment of Constipation-Quality of Life (PAC-QOL) by a researcher's assistant who was not aware of the study groups. Additionally, the demographic survey included some information such as age, sex, marital status, lifestyle (i.e., physical activity level and smoking), and medical history. After reviewing the literature, this questionnaire was prepared and sent to experts to confirm its validity.

The CAS was developed by McMillan and Williams (1989) with a reliability of 0.86 and validity of 0.83 [37]. Seyyedrassoli et al. also reported the reliability of 0.98 for this scale [38]. This scale contained eight items, namely, “abdominal distension or bloating,” “change in the amount of gas passed rectally,” “less frequent bowel movement,” “oozing liquid stool,” “rectal fullness or pressure,” “rectal pain with bowel movement,” “small volume of the stool,” and “being unable to pass stool.” The abovementioned items could be scored 0 (no problem), 1 (some problems), or 3 (severe problem). Thus, the total score could range from 0 to 16 that indicated no constipation and severe constipation, respectively [37].

BSFS was designed by Lewis and Heaton [39] and was translated into Persian under PROJECT ID TPT 890266 in 2017. This scale was used to evaluate the seven categories of changes in different bowel habits. For instance, types 1 and 2 showed constipation, types 3 and 4 represented regular bowel movements, and types 5, 6, and 7 showed diarrhea [39].

PAC-QOL was developed by Marquis et al., with acceptable Cronbach's alpha coefficient (0.91) and test-retest reliability (*r* = 0.96, *p* < 0.001) [40]. Nikjooy et al. also evaluated this questionnaire and confirmed its reliability by Cronbach′s alpha = 0.92 (range: 0.72-0.92; *p* < 0.001) [41]. This 28-item questionnaire was used to assess the effects of constipation on the patients' quality of life in the last two weeks. The items were divided into four categories, namely, worries (11 items), physical discomfort (4 items), psychosocial discomfort (8 items), and treatment satisfaction (5 items). The highest and lowest scores of this scale were 140 and 28, respectively [40].

### 2.5. Statistical Analysis

At first, the data were entered into the SPSS 22 software and were assessed for normal distribution. Then, data analysis was performed on codes using descriptive and inferential statistics including chi-square, paired *t*-test, independent *t*-test, and ANOVA. *p* ≤ 0.05 was considered statistically significant.

### 2.6. Ethical Issues

This study was performed after obtaining permission from the Ethics Committee of Shiraz University of Medical Sciences (IR.SUMS.REC.1399.778). The objectives and procedures of the study were explained to the participants and their written informed consent forms were obtained. They were also assured that they could leave the study at any stage.

## 3. Results

In this study, 90 eligible elderly individuals with constipation were assessed based on the inclusion criteria. However, only 79 patients were finally analyzed, because 11 older adults were excluded due to reluctance to participate, suffering from diarrhea, and noncontinuation of the intervention. The mean age of the participants was 67.38 + 3.92 years.

Based on the results presented in Table 1, no significant difference was observed among the three groups in terms of the demographic variables (*p* > 0.05).

The results revealed no significant difference among the three groups in terms of the scores of CAS and BSFS before the intervention. However, a statistically significant difference was observed among the three groups regarding the score of PAC-QOL before the intervention (Tables 2 and 3). Based on the results of the LSD post hoc test, the mean score of constipation was significant in the oral consumption group compared to the abdominal massage group and the control group (*p* < 0.001). In other words, the mean score of CAS significantly decreased from 7.81 ± 2.62 to 1.15 ± 1.08 in the oral consumption group after the intervention (*p* < 0.0003). In the abdominal massage group also, the mean score of CAS significantly decreased from 8 ± 2.57 to 2.26 ± 1.81 after the intervention (*p* < 0.0002). In the control group, the mean score of constipation significantly decreased from 7.73 ± 2.53 to 6 ± 2.74 (*p* < 0.0002) (Table 2). On the other hand, the mean score of quality of life was significant in the oral consumption group compared to the abdominal massage group and the control group (*p* < 0.001). According to the results, the score of quality of life decreased from 75.04 ± 7.66 to 28.15 ± 3.30 in the oral consumption group (*p* < 0.0003), from 81.15 ± 5.15 to 43.26 ± 4.13 in the abdominal massage group (*p* < 0.0001), and from 79.62 ± 4.76 to 62.31 ± 6.40 in the control group (*p* < 0.0008) (Table 2).

The analysis of the subitems of CAS showed a significant difference among the three groups regarding “abdominal distension or bloating,” “change in the amount of gas passed rectally,” “less frequent bowel movements,” “rectal fullness or pressure,” “rectal pain with bowel movement,” “the small volume of stool,” and “unable to pass stool” after the intervention (*p* < 0.05) (Table 4).

The rate of improvement in stool consistency was evaluated by comparing the differences in the three groups' Bristol scores at the beginning and at the end of the intervention. This score was 4.885 ± 1.275 in the oral consumption group, 3.296 ± 0.724 in the abdominal massage group, and 1.308 ± 0.549 in the control group. Based on the results, this score was significantly higher in the oral consumption group than in the other groups (*p* < 0.0003) (Table 3).

The quality of life subgroups including physical discomfort, psychosocial discomfort, anxiety, and satisfaction was significantly different in the oral consumption group compared to the abdominal massage and control groups (*p* < 0.05) (Table 5).

## 4. Discussion

The study results demonstrated that the administration of sweet almond oil significantly decreased the constipation score and improved stool consistency in the elderly participants. In the same line, Zeinalian et al. reported that the oral administration of sweet almond oil could treat constipation in old patients [17]. Overall, the effectiveness of oral consumption of sweet almond oil and other oils has been expressed in Persian medicine, especially for the treatment of constipation [18, 42, 43].

In the present study, the constipation score decreased significantly in the abdominal massage group after the intervention. However, this reduction was more prominent in the oral consumption group. Additionally, despite the significance of stool consistency, it improved less compared to the oral consumption group. Generally, abdominal massage is very popular due to its inexpensiveness, lack of specific complications, and lack of interference with other treatments [44]. Çevik et al. indicated that abdominal massage treated constipation and stool consistency in elderly individuals [11]. Faghihi et al. also revealed that abdominal massage effectively treated constipation in elderly people [30]. Abdominal massage with oils can be applied as a complementary method in the management of constipation especially in elderly [45].

The current study findings revealed a decrease in constipation and an improvement in stool consistency in the control group, which might be attributed to the administration of medicines prescribed by gastroenterologists. However, the decrease in constipation was more prominent in the two other groups.

The present study results showed a significant increase in the quality of life of the elderly participants in the oral consumption group compared to the abdominal massage and control groups after the intervention. Many studies have revealed a strong correlation between cured constipation and quality of life [10, 13, 46]. For instance, Turan and Atabek reported that abdominal massage played a vital role in improving patients' quality of life [10]. Okuyan and Bilgili also stated that abdominal massage training for eight weeks increased the quality of life of the older adults living in nursing homes [13].

All in all, nonsurgical methods and complementary medicine such as use of vegetable oils and abdominal massage can provide more treatment options for patients and healthcare providers [47].

In this study, use was made of conventional vegetable oils in Persian medicine, which could be regarded as one of the major strengths of the research. However, one of the study limitations was the COVID-19 pandemic, which resulted in quick group implementation of the project. Another study limitation was that the participants' defecation characteristics were gathered from their own statements, and observational evaluations were not performed.

## 5. Conclusion

The study findings demonstrated that the oral administration of sweet almond oil and abdominal massage using sweet almond oil were effective in the treatment of constipation and improvement of the quality of life among the elderly individuals. Due to the high prevalence of constipation in the elderly population and its adverse effects on the quality of life and other health indicators, this simple, inexpensive, and noninvasive method is recommended to be applied to control constipation. Moreover, further studies are suggested to evaluate the effectiveness of sweet almond oil in constipation among older adults with other clinical conditions.

## Figures and Tables

**Table 1:** Comparison of the three groups regarding the demographic characteristics.

Variable	Control *N* (%)		Abdominal massage with almond oil^∗^*N* (%)	Oral consumption of almond oil^∗∗^*N* (%)	Test results
Sex	Female	12 (46.2)	14 (51.9)	15 (57.7)	*χ* ^2^ = 0.693
	Male	14 (53.8)	13 (48.1)	11 (42.3)	*p* = 0.721
Education	Illiterate	0 (0)	0 (0)	2 (7.7)	*χ* ^2^ = 9.834
	Primary school	2 (7.7)	3 (11.1)	2 (7.7)	*p* = 0.639
	Middle school	2 (7.7)	5 (18.5)	4 (15.4)	
	High school	16 (61.5)	13 (48.1)	9 (34.6)	
	Associate degree	3 (11.5)	3 (11.1)	6 (23.1)	
	Bachelor's degree	3 (11.5)	2 (7.4)	3 (11.5)	
	Master's and higher degrees	0 (0)	1 (3.7)	0 (0)	
Marital status	Married	19 (73.1)	16 (59.3)	18 (69.2)	*χ* ^2^ = 1.674
	Single	2 (7.7)	4 (14.8)	2 (7.7)	*p* = 0.957
	Divorced	2 (7.7)	3 (11.1)	3 (11.5)	
	Widowed	3 (11.5)	4 (14.8)	3 (11.5)	
Job	Worker	2 (7.7)	4 (14.8)	2 (7.7)	*χ* ^2^ = 4.689
	Employee	3 (11.5)	3 (11.1)	2 (7.7)	*p* = 0.976
	Retired	6 (23.1)	6 (22.2)	10 (38.5)	
	Self-employed	3 (11.5)	2 (7.4)	1 (3.8)	
	Farmer	1 (3.8)	2 (7.4)	1 (3.8)	
	Homemaker	9 (34.6)	9 (33.3)	9 (34.6)	
	Military staff	2 (7.7)	1 (3.7)	1 (3.8)	
Smoking	Yes	11 (42.3)	9 (33.3)	9 (34.6)	*χ* ^2^ = 0.532
	No	15 (57.7)	18 (66.7)	17 (65.4)	*p* = 0.838
Exercising	Yes	10 (38.5)	14 (51.9)	7 (26.9)	*χ* ^2^ = 3.462
	No	16 (61.5)	13 (48.1)	19 (73.1)	*p* = 0.195
Age	Mean ± SD	66.62 ± 3.56	67.33 ± 3.89	68.19 ± 4.26	*F* = 1.056 *p* = 0.353

**Table 2:** Comparison of the three groups regarding the mean scores of the study variables.

Group	Variable (mean ± SD)				*p* value^∗^	
	CAS		QOL			
	Before	After	Before	After	CAS	QOL
Control	7.73 ± 2.53	6 ± 2.74	79.62 ± 4.76	62.31 ± 6.40	*t* = 10.093 *p* < 0.0002	*t* = 13.212 *p* < 0.0008
Abdominal massage with almond oil	8 ± 2.57	2.26 ± 1.81	81.15 ± 5.15	43.26 ± 4.13	*t* = 24.298 *p* < 0.0002	*t* = 39.056 *p* < 0.0001
Oral consumption of almond oil	7.81 ± 2.62	1.15 ± 1.08	75.04 ± 7.66	28.15 ± 3.30	*t* = 18.747 *p* < 0.0003	*t* = 27.389 *p* < 0.0003
*p* value^∗∗^	*F* = 0.077 *p* = 0.926	*F* = 42.219 *p* < 0.0004	*F* = 7.407 *p* = 0.001	*F* = 331.889 *p* < 0.0002		

^∗^Paired *t*-test; ^∗∗^One-way ANOVA.

**Table 3:** Comparison of the three groups regarding the Bristol score.

Variables	Control *N* (%)		Abdominal massage with almond oil *N* (%)	Oral consumption of almond oil *N* (%)	Test results
Bristol.pre	Type 1	15 (57.7)	10 (37)	19 (73.1)	**χ** ^2^ = 11.837 *p* = 0.081
	Type 2	6 (23.1)	10 (37)	3 (11.5)	
	Type 3	4 (15.4)	7 (25.9)	3 (11.5)	
	Type 4	1 (3.8)	0 (0)	0 (0)	
	Type 5	0 (0)	0 (0)	1 (3.8)	
Bristol.post	Type 1	1 (3.8)	0 (0)	0 (0)	**χ** ^2^ = 76.244 *p* < 0.0003
	Type 2	8 (30.8)	0 (0)	0 (0)	
	Type 3	10 (38.5)	0 (0)	0 (0)	
	Type 4	6 (23.1)	9 (33.3)	0 (0)	
	Type 5	0 (0)	10 (37)	4 (15.4)	
	Type 6	1 (3.8)	2 (7.4)	8 (30.8)	
	Type 7	0 (0)	6 (22.2)	14 (53.8)	

**Table 4:** Comparison of the three groups regarding the eight items of CAS before and after the intervention.

Item	Group	Control	Abdominal massage with almond oil	Oral consumption of almond oil	*p* value^∗^
		Mean ± SD	Mean ± SD	Mean ± SD	
Abdominal distension or bloating	Before	1.27 ± 0.533	1.26 ± 0.526	1.19 ± 0.849	0.94
	After	0.96 ± 0.445	0.37 ± 0.492	0.23 ± 0.43	<0.0001
Change in the amount of gas passed rectally	Before	1 ± 0.49	0.96 ± 0.759	0.92 ± 0.744	0.81
	After	0.92 ± 0.56	0.26 ± 0.447	0	<0.0001
Less frequent bowel movement	Before	1.23 ± 0.652	0.78 ± 0.698	1.08 ± 0.744	0.02
	After	0.88 ± 0.711	0.15 ± 0.362	0.08 ± 0.272	<0.0001
Oozing liquid stool	Before	0.04 ± 0.196	0.04 ± 0.192	0.15 ± 0.368	0.97
	After	0	0	0	1
Rectal fullness or pressure	Before	0.81 ± 0.749	1 ± 0.877	1.12 ± 0.588	0.42
	After	0.62 ± 0.571	0.3 ± 0.465	0.15 ± 0.368	0.03
Rectal pain with bowel movement	Before	0.73 ± 0.667	1.26 ± 0.594	0.88 ± 0.588	0.005
	After	0.69 ± 0.549	0.33 ± 0.48	0.15 ± 0.368	0.01
The small volume of stool	Before	1.5 ± 0.51	1.63 ± 0.492	1.42 ± 0.578	0.34
	After	1.23 ± 0.43	0.67 ± 0.48	0.38 ± 0.496	<0.0001
Unable to pass stool	Before	1.15 ± 0.543	1.07 ± 0.55	1.04 ± 0.528	0.59
	After	0.69 ± 0.471	0.19 ± 0.396	0.15 ± 0.368	<0.0001

^∗^Kruskal-Wallis test.

**Table 5:** Comparison of the three groups regarding the eight items of quality of life before and after the intervention.

Item	Group	Control	Abdominal massage with almond oil	Oral consumption of almond oil	*p* value
		Mean ± SD	Mean ± SD	Mean ± SD	
Physical discomfort	Before	13.1 ± 1.8	13.1 ± 1.4	12.4 ± 1.8	0.205
	After	9.4 ± 1.6	4.9 ± 1.4	2.8 ± 2.1	<0.0001
Psychosocial discomfort	Before	26.1 ± 2.9	26.5 ± 2.5	24.6 ± 3.7	0.13
	After	19.03 ± 3.2	11.2 ± 1.6	4.11 ± 2.1	<0.0001
Anxiety	Before	35.9 ± 2.9	36.8 ± 2.2	33.5 ± 4.2	0.001
	After	26.03 ± 2.9	14.3 ± 1.4	6.0 ± 2.1	<0.0001
Satisfaction	Before	4.3 ± 1.4	4.8 ± 1.8	4.3 ± 1.4	0.42
	After	7.8 ± 2.07	12.7 ± 1.9	15.1 ± 1.4	<0.0001
PAC-QOL total	Before	79.62 ± 4.76	81.15 ± 5.15	75.04 ± 7.66	0.01
	After	62.31 ± 6.40	43.26 ± 4.13	28.15 ± 3.30	<0.0003

## Data Availability

The datasets used and analyzed during the current study are available from the corresponding author on reasonable request.
